# A cross-sectional study on workplace experience: a survey of nurses in Quebec, Canada

**DOI:** 10.1186/s12960-019-0358-4

**Published:** 2019-03-14

**Authors:** Marie-Annick Gagné, Carl-Ardy Dubois, Alexandre Prud’Homme, Roxane Borgès Da Silva

**Affiliations:** 1 Faculty of Nursing, University of Montreal, Montreal, Canada; 2 Public Health Research Institute, University of Montreal, Montreal, Canada; 3 School of Public Health, Department of Management, Evaluation and Health Policy, University of Montreal, 7101 Avenue du Parc, Montréal, C.P. 6128, succursale Centre-ville, Montreal, QC H3C 3J7 Canada

**Keywords:** Nursing practice, Work organization, Workforce research, Workplace experience, Nursing administration

## Abstract

**Background:**

Nurses play a significant role in healthcare systems. Their workplace experience can have an impact not only on nurses themselves, but also on patients and organizations, particularly in terms of quality of care and performance. Despite the importance of this experience, it remains an ambiguous concept with varying interpretations. Current studies do not fully capture its complexity, as its multiple dimensions are often considered in isolation. As such, developing a portrait of nurses’ workplace experience that integrates its multiple dimensions can provide decision-makers with better indications regarding what levers can be mobilized to generate positive results for nurses, patients, and organizations.

**Aim:**

To identify profiles of nurses’ workplace experience in Quebec, Canada.

**Design:**

Cross sectional.

**Methods:**

In April 2017, 891 nurses participated in this study by completing a self-administered questionnaire. Four dimensions of nurses’ workplace experience were measured: resources available to them in their workplace, personal resources, demands (psychological and physical) placed on them, and outcomes associated with their work. Descriptive and factorial analyses were performed.

**Results:**

Three profiles of nurses’ workplace experience emerged from the factorial analyses: nurses in distress, nurses in moderately positive situations, and nurses in positive situations.

**Conclusion:**

The study identified profiles of nurses’ workplace experience that were differentiated based on nurses’ access to workplace resources, the demands of their work, and outcomes. Healthcare managers can use the results to improve the quality of nurses’ workplace experience by improving access to structural work resources and alleviating psychological demands.

## Introduction

In healthcare systems, nurses play an essential role, not only because they are the largest segment of the workforce, but also because they perform a range of critical functions at all points along the continuum of care and services. While the issue of nursing staff availability is often the main focus of attention, their workplace experience can have an impact on both the nursing workforce and the quality of care provided by nurses [1–3].

However, available data suggest a number of concerns related to nurses’ workplace experience. Workplace dissatisfaction and health problems are among the symptoms indicative of a workplace experience that is perceived negatively by nurses [4, 5]. Several surveys have shown that nurses are a group of workers particularly vulnerable to illness, injury, and violence in the workplace [5, 6]. For health organizations, indicators of this adverse workplace experience for nurses are high absenteeism [5, 7] and high turnover [5, 8]. These problems engender additional costs for these organizations, associated with the use of external labour, overtime [9], and time spent recruiting and training new staff [8]. Negative workplace experience has also been associated with poorer quality of care and increased incidence of adverse events among patients [4, 10].

Despite the importance of nurses’ workplace experience and its many implications, it remains a complex and ambiguous concept open to diverse interpretations. The current literature, while abundant, provides only fragmented and heterogeneous views of this concept, either because its multiple dimensions are more often considered in isolation rather than together, or because the orientations taken often differ from one study to another. As a result, managers lack clear guidelines on the levers that can be mobilized to improve nurses’ workplace experience and thereby generate positive effects on patients and organizations in terms of quality of care and performance. Producing a comprehensive portrait of the workplace experience of nurses and implementing improvement strategies therefore requires a conceptualization that takes into account the multidimensional nature of the concept. The aim of this study conducted in Quebec was to review the different perspectives adopted in the literature to assess nurses’ workplace experience and to identify profiles of nurses’ workplace experience based on the Job Demands-Resources model [11]. The paper is structured in five sections. The first one presents the different perspectives used in the literature to characterize workplace experience. In the second section, we present the framework we used for this study. The following sections present methods, results, and discussion.

## Background

The literature presents three different perspectives that are often used in the study of nurses’ workplace experience. The first is a structural perspective that emphasizes nurses’ relationship with their work environment. This perspective is in line with Kanter’s work on structural empowerment. According to Kanter’s (1977) theory, empowerment is the process by which an organization optimizes performance by ensuring the worker is well equipped, informed, and supported [12]. According to Laschinger et al., empowerment factors include nurses’ access to information, a variety of supports, operating resources, and professional development opportunities [13]. These factors offer nurses the ability to mobilize different resources present in their work to facilitate its achievement [14, 15]. The resulting structural empowerment generates a psychological response in which nurses feel more motivated, competent, and in control [16, 17]. Laschinger et al.’s model also showed a strong association between empowerment, stress, and satisfaction [13]. This perspective thus emphasizes structural resources as an important dimension of nurses’ workplace experience and links structural resources to outcomes. Recent empirical studies, such as those from the RN4CAST project, have adopted this perspective by focusing on organizational characteristics such as staffing and resource adequacy [18], with increasing evidence showing that these are key factors that impact on nurse retention, burnout among nurses, and patient outcomes.

The second perspective refers to work processes and emphasizes nurses’ relationship with the organization of their work. These work processes are of various kinds. Echoing sociotechnical models [19], they encompass both technical (procedures, tools, technical organization of work) and social (human relations) systems. Work processes put forward in this model are autonomy and the need for recognition and support. According to Trist, they contribute to increased work engagement [19]. Similarly, the job characteristics model of Hackman and Oldham (1975) focuses on variety of skills used, task identity, meaning of work, autonomy, and feedback as work processes [20]. When work organization includes these processes, workers experience a variety of psychological states associated with different outcomes in terms of satisfaction and motivation. Edgar (1999) adjusted this model for use in nursing [21]. The resulting job characteristics model demonstrated that autonomy and communication with co-workers were associated with nurse satisfaction and motivation [21]. In this perspective on nurses’ workplace experience, various elements of work processes, such as decision-making autonomy, clear division of roles, and support and feedback, are associated with several outcomes, including job satisfaction and engagement. Many recent studies have examined these work processes using the Practice Environment Scale, which includes nurse manager ability, nurse participation in hospital affairs, nursing foundation for quality of care, nurse–doctor relationship, and cultural values [22].

The third perspective on nurses’ workplace experience refers to outcomes, i.e., the consequences nurses may experience as a result of unfavourable work environments or organizations. These outcomes are conceptualized in terms of health, satisfaction, engagement, and intention to quit. In terms of health, nurses’ workplace experience is often related to stress and emotional exhaustion or burnout. Stress is defined as individuals’ response to the perceived gap between the demands placed upon them and their capacities [23]. When stress exposure becomes chronic, it can lead to burnout [24]. Although these results can be modulated by personal characteristics, they are mainly influenced by the extent to which workers have access to structural work resources and work processes. Studies have identified several features of the environment and work organization that are likely to lead to stress and burnout among nurses, such as understaffing and lack of support from superiors and colleagues [23–26]. Workplace experience is also conceptualized in terms of job satisfaction, i.e., alignment between nurses’ needs and their capacity to satisfy those needs through their work [27]. While this satisfaction may be intrinsic and thus self-reported, it also depends on the work environment and its organization. Several researchers surveying nurses have identified workplace factors that lead to dissatisfaction: understaffing [28]; lack of recognition, support, accessibility, communication, and flexibility on the part of superiors [29]; and poor quality of relations with colleagues [30]. Engagement refers to individuals’ personal involvement in the organization. Work engagement results from nurses’ having access to various resources, such as career advancement opportunities and support from superiors [31], which echoes the factors in Kanter’s structural empowerment model. However, nurses’ engagement with work can also be influenced by work processes such as decision-making autonomy and recognition of efforts [32–34]. Finally, while intention to quit is often related to job satisfaction [8, 27, 35], this outcome also depends both on nurses’ access to structural resources and on work processes. Access to professional development opportunities and quality of relationships with superiors also affect nurses’ intention to leave their jobs [27]. This last perspective highlights the key outcomes used to study nurses’ workplace experience, such as stress, emotional exhaustion, satisfaction, engagement, and intention to quit. However, interpreting outcomes alone is not sufficient to fully understand nurses’ workplace experience, as these outcomes may be influenced by both work structure and processes. To obtain an accurate reading, all dimensions must be integrated into the study of this concept.

### Framework

This study was based on the Job Demands-Resources model [11], which integrates the three perspectives described above. This model assumes each occupation has characteristics that may cause strain. These can be classified into two broad categories: work demands and work resources [11]. Demands correspond to the physical and psychosocial aspects of work that require effort and therefore energy expenditure [36]. Resources are associated with the elements of work that enable individuals to accomplish their tasks, reduce the demands placed on them, and stimulate their development [36]. Personal resources can have the same effect as work resources, by reducing the negative impact of demands. As such, the sense of self-efficacy is identified in the literature as a personal resource, defined as individuals’ conviction that they have control over various situations or requirements of their environment by implementing actions that help them adapt [37, 38]. When resources exceed demands, workers may feel more motivated and experience a greater sense of engagement [36]. Conversely, when resources are insufficient to cope with excessive demands, workers may suffer from burnout, develop health problems, and be more dissatisfied [11].

**Fig. 1:**
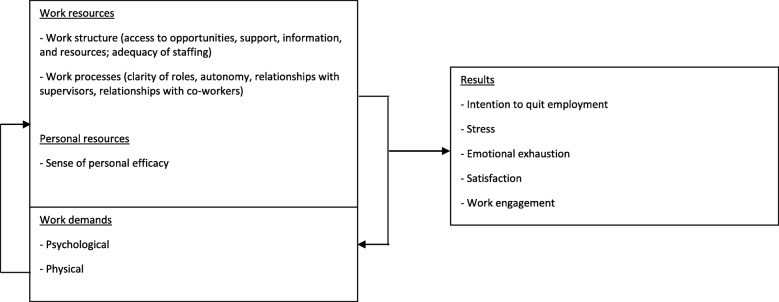
Conceptual model of nurses’ workplace experience adapted from Bakker and Demerouti’s (2007) Job Demands-Resources model

According to this model, nurses’ workplace experience (Fig. 1) encompasses four dimensions. The first refers to resources available to nurses in their work. These can be structural in nature or linked to work processes. Extending these resources, the second dimension refers to personal resources. The third consists of the demands placed on nurses. The balance between access to resources and demands influences the fourth dimension, which refers to outcomes associated with the work.

## The study

### Aim

The purpose of this study was to produce profiles of nurses’ workplace experience in Quebec, a province of Canada.

### Design

This cross-sectional quantitative study was based on a descriptive non-experimental design.

### Participants

The Ordre des infirmières et infirmiers du Québec (OIIQ) took itself charge of the extraction of a stratified sample according to our requests. In 2016–2017, 74 469 nurses were registered in the province of Quebec. However, we asked for a sample among registered nurses practising in a clinical area in the province of Quebec (*N* = 62 084), as well ensuring a representative proportion of the four fields of practice (physical healthcare, mental healthcare, critical care, and primary care). Thus, nurses whose main activity was teaching, management, or research were excluded from the sampling. Our objective was to get a margin of error less than 4%, using a confidence level of 95%. Because the usual response rate experimented by the OIIQ for this kind of study was around 10%, we calculated that we needed at least 515 respondents to achieve our goal. Thereby, the OIIQ extracted a stratified random sample of 5161 nurses. Among the 5161 nurses that received an invitation to participate in our study, 891 completed the questionnaire (response rate = 17%), which gives us a margin of error of 3.3%.

### Data collection

Data were collected using a self-administered French questionnaire sent electronically to the selected nurses during the month of April 2017. The variables and the instruments used are presented in Table 1.

**Table 1:** Measured variables and instruments

Dimensions	Measured variable	Instruments	α*
Structural resources of work	Access to opportunities, information, support, and resources	*Conditions of Work Effectiveness Questionnaire II* (Laschinger et al., 2001) [13]	0.85
	Adequacy of staffing	*Staffing and Resource Adequacy* subscale of the *Pratice Environment Scale of the Nursing Work Index* (Lake, 2002) [39]	0.80
Resources related to work processes	Clarity of roles	*Role Ambiguity* subscale of the *Role Conflict and Ambiguity Scale* (Rizzo, House, & Lirtzman, 1970) [40]	0.82
	Autonomy	Items concerning autonomy in the *Job Diagnostic Survey* (*JDS*) (Hackman & Oldham, 1975) [20]	0.86
	Relationship with supervisor	Supervisor support subscale of the EQCOTESST survey (Vézina et al., 2011) [41]	0.83
	Relationship with co-workers	Co-worker support subscale of the EQCOTESST survey (Vézina et al., 2011) [41]	0.66
Personal resources	Sense of personal efficacy	*General Self-Efficacy Scale* (Jerusalem & Schwarzer, 1995) [42]	0.87
Work demands	Physical demands of work	General index of cumulative physical constraints of work in the EQCOTESST survey (Vézina et al., 2011) [41]	0.82
	Psychological demands of work	Psychological demands subscale of the EQCOTESST survey (Vézina et al., 2011) [41]	0.72
Outcomes	Intention to quit employment	Items based on the theory of Mobley, Horner, and Hollingsworth (1978) [43]	0.90
	Stress	*Perceived Stress Scale* (Cohen, Kamarck, & Mermelstein, 1983) [44]	0.85
	Emotional exhaustion	*Emotional Exhaustion* subscale of the *Maslach Burnout Inventory* (Maslach & Jackson, 1981) [45]	0.90
	Work satisfaction	Satisfaction subscale of the COPSOC-V3 (Dupret, Bocéréan, Teherani, & Feltrin, 2012) [46]	0.75
	Work engagement	*Utrecht Work engagement Scale* (Schaufeli, Salanova, Gonxales-Roma & Bakker, 2002) [47]	0.92

* Cronbach’s alpha coefficient

### Validity, reliability, and rigour

The instruments used in this study were all of acceptable validity and reliability. Cronbach’s alpha coefficients (*α*) ranged from 0.66 to 0.92 (Table 1). The tools were previously validated in French in previous studies.

### Ethical considerations

This study was approved by the University of Montreal’s health research ethics committee. To access the questionnaire, the selected nurses first had to read the information and consent form and then agree to participate in the study. Nurses’ participation was anonymous to protect data confidentiality.

## Data analysis

Following observation of distributions, the variables were transformed to produce three ordinal categories (low, medium, high), except for the satisfaction variable (low, high). The data analysis method consisted of two steps. In the first, multiple correspondence analysis (MCA) was used to identify the most significant data structures (factorial axes). In a second step, ascending hierarchical classification (AHC) was used to construct the classification tree structure [48]. To select the final classification, the tree structure and inertia quotients (interclass/total inertia) were observed. Cramer’s contingency coefficient (CCC) was used to measure the strength of association between variables and nurses’ workplace experience profiles [49]. A CCC equal to or lower than 0.3 indicates a low to moderate associative strength, whereas a CCC equal to or greater than 0.50 indicates a strong associative strength [50]. SPSS 23® and SPAD 8® were used for data analysis.

## Results

### Description of the participants

Age group categories were generally well represented across the 891 participants (18–25 years = 13.9%; 26–35 years = 31.2%; 36–45 years = 26.0%; 46–55 years = 19.9%), with the exception of those aged 56 and over (56–65 years = 8.4%; 66 years and over = 0.6%). The majority of participants had a university undergraduate degree (bachelor’s degree = 63.0%) and worked the day shift (59.8%).

### Description of the measured variables

The results showed that a large majority of nurses perceived all measured variables as moderate (Table 2). However, nurses’ perceptions of access to career development and advancement opportunities were quite high (65.0%), as were their satisfaction (79.1%) and engagement (55.8%). In addition, high proportions of nurses indicated that the physical demands of their work were low (78.0%) and that their intention to leave their job was low (64.3%).

**Table 2:** Description of the measured variables

Dimensions	Variables	Scores	Total % (*n* = 891)	Nurses in distress % (*n* = 231)	Nurses in moderately favourable situations % (*n* = 446)	Nurses in favourable situations % (*n* = 214)	CCC*
Structural resources of work	Access to information	Low	35.4	66.7	29.4	14.0	0.348
		Medium	45.3	27.3	56.0	42.5	
		High	19.3	6.0	14.6	43.5	
	Access to opportunities	Low	3.5	12.6	0.2	0.5	0.348
		Medium	31.5	53.3	26.5	18.7	
		High	65.0	34.2	73.3	80.8	
	Access to support	Low	41.9	77.9	36.1	15.0	0.439
		Medium	42.9	21.7	56.1	38.3	
		High	15.2	0.4	7.8	46.7	
	Access to operation resources	Low	30.0	68.8	21.7	5.1	0.497
		Medium	52.0	27.3	70.4	40.7	
		High	18.0	3.9	7.9	54.2	
	Adequacy of staffing	Low	23.8	52.4	15.7	9.8	0.405
		Medium	63.3	39.8	80.7	52.3	
		High	12.9	7.8	3.6	37.9	
Resources related to work processes	Clarity of roles	Low	11.3	31.6	5.6	1.4	0.424
		Medium	41.2	50.2	52.5	7.9	
		High	47.5	18.2	41.9	90.7	
	Autonomy	Low	10.3	33.3	2.5	1.9	0.432
		Medium	42.4	52.8	50.9	13.5	
		High	47.3	13.9	46.6	84.6	
	Relationship with supervisor	Low	25.8	57.6	18.6	6.5	0.416
		Medium	56.2	36.8	71.3	45.8	
		High	18.0	5.6	10.1	47.7	
	Relationship with co-workers	Low	2.7	6.9	1.4	0.9	0.213
		Medium	53.1	55.0	62.3	31.8	
		High	44.2	38.1	36.3	67.3	
Personal resources	Sense of personal efficacy	Low	17.3	35.1	14.6	3.7	0.273
		Medium	37.8	33.3	46.4	24.8	
		High	44.9	31.6	39.0	71.5	
Work demands	Physical demands	Low	78.0	69.3	80.7	81.8	0.115
		Medium	14.1	18.1	11.2	15.9	
		High	7.9	12.6	8.1	2.3	
	Psychological demands	Low	9.0	2.6	3.6	27.1	0.349
		Medium	53.8	29.4	65.0	59.5	
		High	37.2	68.0	31.4	13.4	
Outcomes	Intention to quit employment	Low	64.3	27.7	72.9	86.0	0.349
		Medium	18.7	29.9	17.9	8.4	
		High	17.0	42.4	9.2	5.6	
	Stress	Low	9.7	9.9	7.2	14.5	0.133
		Medium	77.4	68.4	81.8	78.0	
		High	12.9	21.7	11.0	7.5	
	Emotional exhaustion	Low	43.7	8.7	45.1	78.0	0.415
		Medium	32.0	32.0	38.8	18.2	
		High	24.3	59.3	16.1	3.8	
	Work satisfaction	Low	20.9	59.3	7.6	7.0	0.559
		High	79.1	40.7	92.4	93.0	
	Work engagement	Low	6.1	19.0	1.8	0.9	0.308
		Medium	38.1	53.3	38.3	21.5	
		High	55.8	27.7	59.9	77.6	

*Cramer’s contingency coefficient

### Nurse workplace experience profiles

**Fig. 2:**
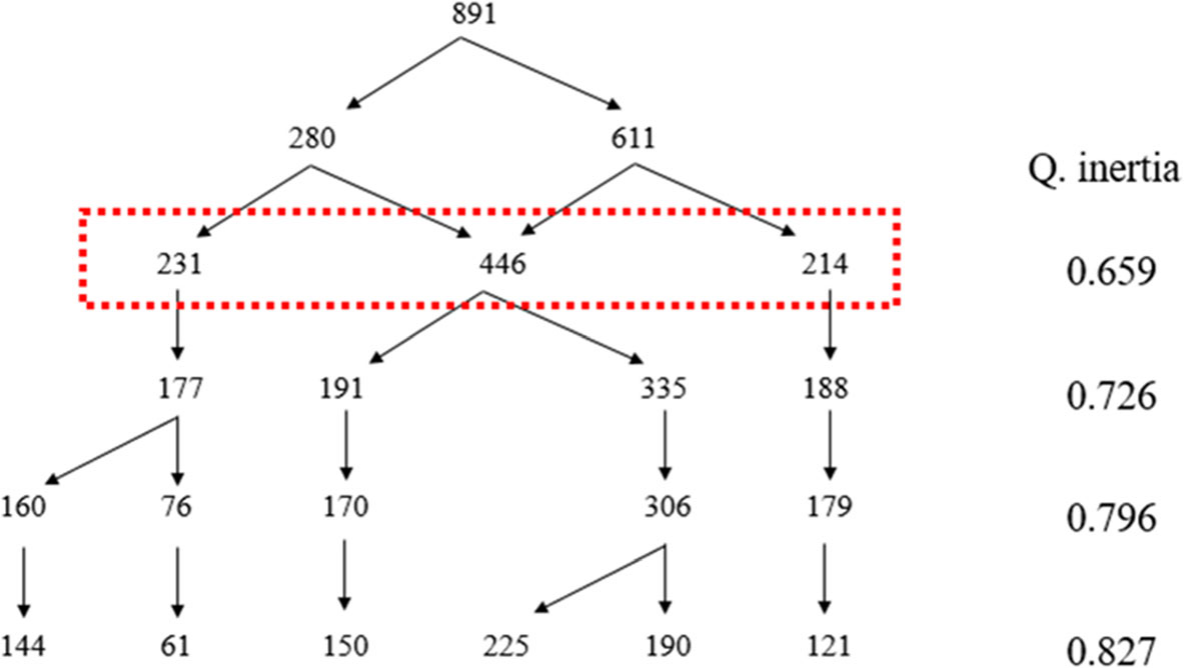
Tree structure and inertia quotients

In the first stage of data analysis, the first three axes were selected, as they accounted for 98% of the variance. The tree structure produced by AHC and inertia quotients is shown in Fig. 2. From the inertia quotients (Q. inertia), a plateau of sorts emerged from the four-profile classification, but the three-profile classification was retained because it was easier to interpret.

The three profiles of nurses’ workplace experience are labeled as follows: nurses in distress (*n* = 231), nurses in moderately positive situations (*n* = 446), and nurses in positive situations (*n* = 214). Table 2 presents the characteristics of the three profiles.

The nurses-in-distress profile (*n* = 231) comprised just over one quarter of the total sample (25.9%). This workplace experience profile was characterized by nurses who perceived themselves as having low to moderate access to workplace resources, high psychological demands, and negative outcomes. With respect to resources, access to structural work resources was low for all variables except for access to professional development opportunities, which was considered moderate by the majority of nurses in this profile. For resources related to work processes, access was moderate, except for the supervisor relationship variable, perceived as low by most nurses in this first workplace experience profile. In terms of workplace demands, psychological demands were deemed high and physical ones rather low. However, of the three profiles, this one contained the most nurses who rated the physical demands of their work as high. Outcomes were generally reported as negative. Even so, nurses in this profile remained moderately engaged with their work.

The nurses-in-moderately-positive-situations profile (*n* = 446) included half of the nurses in the sample (50.1%). Nurses in this profile were characterized by a moderate perception of all variables related to access to workplace resources and work demands, as well as by rather positive outcomes. Access to structural work resources was rated as moderate across all variables, except for access to professional development opportunities, considered high by the majority of nurses in this profile. As for resources related to work processes, access was moderate for all variables. Psychological demands were also considered moderate. Despite this moderate trend, overall outcomes were rather positive. These nurses’ job satisfaction and engagement were high, while burnout and intention to quit were low.

The nurses-in-a-positive-situation profile (*n* = 214) was the smallest, containing less than one quarter of the sample (24.0%). This last profile was characterized by high access to workplace resources, low to moderate demands, and positive outcomes. Access to structural work resources and work process resources was high for all variables, except for staffing adequacy, which was moderate. However, of the three profiles, this group rated staffing most favourably. With respect to work demands, the majority of nurses in this profile considered that physical demands were low and psychological demands moderate. Outcomes were clearly positive: high job satisfaction and engagement, with low levels of burnout and intention to quit.

The sociodemographic and professional characteristics of nurses in the three profiles are presented in Tables 2 and 3. Practice settings and shifts were significantly associated with the profiles. Nurses working in primary care were more prevalent in the positive situation profile. Nurses working evening and night shifts were more prevalent in the in-distress profile.

**Table 3:** Profiles of nurses’ workplace experience by sociodemographic characteristics

			Profile			
			In distress	In moderately positive situations	In positive situations	Total
			(*n* = 231)	(*n* = 446)	(*n* = 214)	(*n* = 891)
Age group	18–25 (*n* = 124)	% Row	24.2	53.2	22.6	100.0
		% Column	13.0	14.8	13.1	13.9
	26–35 (*n* = 278)	% Row	25.5	51.1	23.4	100.0
		% Column	30.7	31.8	30.4	31.2
	36–45 (*n* = 232)	% Row	26.7	45.7	27.6	100.0
		% Column	26.8	23.8	29.9	26.0
	46–55 (*n* = 177)	% Row	28.8	46.9	24.3	100.0
		% Column	22.1	18.6	20.1	19.9
	56 and over (*n* = 80)	% Row	21.3	61.3	17.5	100.0
		% Column	7.4	11.0	6.5	9.0
Education level	College diploma (*n* = 254)*	% Row	28.7	53.1	18.1	100.0
		% Column	31.6	30.3	21.5	28.5
	Bachelor’s degree (*n* = 558)*	% Row	24.4	48.9	26.7	100.0
		% Column	58.9	61.2	69.6	62.6
	Master’s or doctoral degree (*n* = 54)	% Row	27.8	46.3	25.9	100.0
		% Column	6.5	5.6	6.5	6.1
	Other (*n* = 25)	% Row	28.0	52.0	20.0	100.0
		% Column	3.0	2.9	2.3	2.8

*significant difference between profiles of work experience (*p* ≤ .05)

## Discussion

The aim of this study was to identify profiles of nurses’ workplace experience in the Quebec health and social services network. To our knowledge, this is the first attempt to classify nurses according to workplace experience. The multidimensional approach used to construct the profiles took into account the complex and dynamic nature of nurses’ workplace experience.

In this study, nurses’ workplace experience was characterized according to three profiles differentiated by accessibility of various workplace resources for nurses, intensity of demands, and outcomes (Table 4). The results suggest workplace experience can be perceived differently by considering the dimensions it encompasses and the framework proposed earlier. The nurses-in-distress profile indicated a somewhat negative perception of workplace experience. These nurses had limited access to workplace resources but faced high psychological demands. According to Khamisa et al., nurses whose responsibilities increase in a context of limited resources are particularly prone to stress, burnout, and dissatisfaction [51]. The outcomes measured for the nurses-in-distress profile indicated they were mostly dissatisfied with their work and their emotional exhaustion was high. These results are consistent with those of Aiken et al., whose study of North American and European nurses [17] demonstrated a significant association between access to a variety of resources (staffing adequacy, manager support, relations with colleagues) and satisfaction. The most dissatisfied nurses were more likely to be facing austere administrative measures coupled with a high workload [51]. Hansen et al.’s study of Swedish nurses also highlighted the effects, in terms of burnout, of imbalance between low access to workplace resources and high intensity of demands [52]. Nurses with greater emotional exhaustion reported role conflicts, high workload, and low autonomy [52]. In the study by Khamisa et al., staff problems, such as poor nursing management and insufficient human resources, were strongly associated with burnout and dissatisfaction [51]. However, in the present study, despite this rather negative picture of workplace experience, nurses in this first profile remained very engaged in their work.

**Table 4:** Profiles of nurses’ workplace experience by work characteristics

			Profile			
			In distress	In moderately positive situations	In positive situations	Total
			(*n* = 231)	(*n* = 446)	(*n* = 214)	(*n* = 891)
Years of experience	Less than 1 year (*n* = 55)	% Row	34.5	45.5	20.0	100.0
		% Column	8.2	5.6	5.1	6.2
	1–5 years (*n* = 215)	% Row	27.9	52.6	19.5	100.0
		% Column	26.0	25.3	19.6	24.1
	6–10 years (*n* = 149)	% Row	29.5	43.6	26.8	100.0
		% Column	19.0	14.6	18.7	16.7
	11–20 years (*n* = 245)	% Row	21.2	50.6	28.2	100.0
		% Column	22.5	27.8	32.2	27.5
	21–30 years (*n* = 133)	% Row	27.8	47.4	24.8	100.0
		% Column	16.0	14.1	15.4	14.9
	31 years or more (*n* = 94)	% Row	20.2	59.6	20.2	100.0
		% Column	8.2	12.6	8.9	10.5
Practice setting	Physical health care (*n* = 363)	% Row	28.1	50.4	21.5	100.0
		% Column	44.2	41.0	36.4	40.7
	Mental health care (*n* = 101)	% Row	25.7	49.5	24.8	100.0
		% Column	11.3	11.2	11.7	11.3
	Critical care (*n* = 189)*	% Row	28.6	54.5	16.9	100.0
		% Column	23.4	23.1	15.0	21.2
	Primary care (*n* = 238)*	% Row	20.6	46.2	33.2	100.0
		% Column	21.2	24.7	36.9	26.7
Shift	Day (*n* = 533)*	% Row	22.0	50.7	27.4	100.0
		% Column	50.6	60.5	68.2	59.8
	Evening (*n* = 164)	% Row	31.1	48.2	20.7	100.0
		% Column	22.1	17.7	15.9	18.4
	Night (*n* = 103)*	% Row	33.0	52.4	14.6	100.0
		% Column	14.7	12.1	7.0	11.6
	Rotation (*n* = 91)	% Row	31.9	47.3	20.9	100.0
		% Column	12.6	9.6	8.9	10.2

*significant difference between profiles of work experience (*p* ≤ .05)

There was a strong contrast between the nurses-in-distress profile and that of nurses in a positive situation. Nurses in the latter profile had a positive perception of their workplace experience, as their resources were greater in relation to the demands placed on them. The outcomes measured were much more positive than those of the nurses-in-distress profile. The nurses-in-a-positive-situation profile is comparable to studies on Magnet® hospitals, health organizations recognized for their ability to retain nurses. The efforts made by Magnet hospitals to improve and support the nursing practice environment lead to positive effects not only for patients (quality of care, improved mortality rates) and organizations (nursing workforce stability, cost effectiveness), but also for nurses [53]. Numerous studies have shown that the observable characteristics of Magnet hospitals (management leadership, availability of sufficient resources, nurse involvement in decision-making, access to sources of professional support, collaboration with physician colleagues) increase nurses’ satisfaction and reduce burnout rates and intention to quit [54–57]. These attributes of Magnet hospitals represent a set of resources accessible to nurses. Although no Quebec organization has been granted Magnet hospital status, the nurses-in-a-positive-situation profile might be the one approaching this status the most closely, since these nurses rate their access to workplace resources as high, particularly with regard to professional development opportunities, clarity of roles, autonomy, and support from colleagues.

The nurses-in-moderately-positive-situations profile consisted of nurses with moderate perceptions of their workplace experience. The emergence of this profile was not surprising, nor that it was the largest group. Challenges notwithstanding, these nurses were satisfied with their work in general, felt highly engaged, and had little intention of leaving their jobs. As such, there was a somewhat positive trend in the outcomes measured. It is important to note that this was the most heterogeneous of the three profiles. If a classification with more work experience profiles had been selected, some of these nurses would likely have been in another profile. It may be that personal characteristics explain these results, which appear more favourable considering these nurses were not in an optimal situation a priori. In the present study, the sense of personal efficacy, defined as a perception of control and confidence in one’s ability to adapt to the demands of one’s environment [58, 59], may have played a mediating role in the results obtained. Numerous studies have shown that a strong sense of personal efficacy promotes psychological adaptation to highly stressful events, thereby reducing vulnerability to stress and depression [59]. It also increases engagement, well-being [60], and satisfaction [61]. Given the significant proportions of nurses in this middle profile who rated their sense of personal efficacy as moderate (46.4%) and high (39.0%), this personal resource may have had a positive influence on their workplace experience.

These three profiles were differentiated based on nurses’ level of access to workplace resources, psychological demands, and associated outcomes. There was less variation in access to resources related to work processes. This could be due to the relatively similar patterns of nursing organization from one healthcare institution to another in Quebec.

In the present study, the workplace experience profiles were particularly marked by nurses’ access to structural workplace resources. Further analysis could determine whether these resources are therefore more strongly associated with results. Moreover, given the distinctions among different practice settings, it may be relevant to determine whether there is any association between these settings and the different workplace experience profiles. On a practical level, we encourage healthcare managers to question nurses about their workplace experience, as it can affect performance and retention. Using the specific levers identified in this study for improving nurses’ work experience, managers could focus on increasing nurses’ access to workplace resources and on alleviating the psychological demands of the work.

### Study strengths and limitations

To our knowledge, this is the first study in the field of nursing that has identified different profiles of nurses’ workplace experience. These distinctive profiles can be used to better target the elements of nurses’ work that need to be improved to create more supportive situations, thereby generating positive results not only for nurses, but also for patients and organizations.

Limitations mainly had to do with the representativeness of the sample. Only 33% of nurses registered with the OIIQ agree to be approached to participate in surveys. This may result in a sample of nurses who are more inclined to participate in studies or who are more motivated, which may not be consistent with the overall nurse population. In addition, despite the use of stratified random sampling, young nurses were over-represented and nurses with a college diploma were under-represented. The sampling method could have taken these sociodemographic characteristics into account to increase the representativeness of the sample. The *Tests of proportions*, considering the proportions observed in our sample vs those of the nursing population, have shown that that the sampling show differences with the total nursing population in relation to age and level of education. These observations make it worth mentioning being careful with statistical inference and generalization.

Another potential limitation of this study is the current context of Quebec’s healthcare system. Recent restructuring may have altered some nurses’ perceptions of their workplace experience. Several participants who reported that their working conditions were generally difficult pointed, among other things, to the many job cuts that had occurred in their workplaces. However, these historical factors do not appear to have had a significant impact, since the nurses-in-distress profile represented only one quarter of the sample.

## Conclusion

Overall, nurses in this Quebec study considered themselves to be in a moderately positive situation. Nonetheless, a significant proportion described their workplace experience as relatively unfavourable and reported feeling dissatisfied and exhausted. While the nurses-in-distress workplace experience profile does not reflect the majority, it is important to remember that the outcomes measured for nurses (satisfaction, emotional exhaustion, intention to quit) can have a significant impact on patients and organizations. As such, there is reason for concern about this workplace experience profile, and interventions are needed to improve these nurses’ situation. The results highlighted several levers managers can use to improve nurses’ workplace experience and thereby improve nurse retention and the performance of their health organizations. At the same time, efforts should be made to increase nurses’ access to the structural resources of their workplace and to alleviate the psychological demands imposed by their work.
